# Complementary and alternative medical therapy utilization by people with chronic fatiguing illnesses in the United States

**DOI:** 10.1186/1472-6882-7-12

**Published:** 2007-04-25

**Authors:** James F Jones, Elizabeth M Maloney, Roumiana S Boneva, Ann-Britt Jones, William C Reeves

**Affiliations:** 1 Division of Viral and Rickettsial Diseases, Coordinating Center for Infectious Diseases, Centers for Disease Control and Prevention, Atlanta, GA 30333, USA

## Abstract

**Background:**

Chronic fatiguing illnesses, including chronic fatigue syndrome (CFS), pose a diagnostic and therapeutic challenge. Previous clinical reports addressed the utilization of health care provided to patients with CFS by a variety of practitioners with other than allopathic training, but did not examine the spectrum of complementary and alternative medicine (CAM) therapies used. This study was designed to measure CAM therapy use by persons with fatiguing illnesses in the United States population.

**Methods:**

During a random-digit dialing survey to estimate the prevalence of CFS-like illness in urban and rural populations from different geographic regions of the United States, we queried the utilization of CAM including manipulation or body-based therapies, alternative medical systems, mind-body, biologically-based, and energy modalities.

**Results:**

Four hundred forty fatigued and 444 non-fatigued persons from 2,728 households completed screening. Fatigued subjects included 53 persons with prolonged fatigue, 338 with chronic fatigue, and 49 with CFS-like illness. Mind-body therapy (primarily personal prayer and prayer by others) was the most frequently used CAM across all groups. Among women, there was a significant trend of increasing overall CAM use across all subgroups (p-trend = 0.003). All categories of CAM use were associated with significantly poorer physical health scores, and all but one (alternative medicine systems) were associated with significantly poorer mental health scores. People with CFS-like illness were significantly more likely to use body-based therapy (chiropractic and massage) than non-fatigued participants (OR = 2.52, CI = 1.32, 4.82). Use of body-based therapies increased significantly in a linear trend across subgroups of non-fatigued, prolonged fatigued, chronic fatigued, and CFS-like subjects (p-trend = 0.002). People with chronic fatigue were also significantly more likely to use body-based therapy (OR = 1.52, CI = 1.07, 2.16) and mind-body (excluding prayer) therapy than non-fatigued participants (OR = 1.73, CI = 1.20 – 2.48).

**Conclusion:**

Utilization of CAM was common in fatiguing illnesses, and was largely accounted for by the presence of underlying conditions and poor physical and mental health. Compared to non-fatigued persons, those with CFS-like illness or chronic fatigue were most likely to use body-based and mind-body therapies. These observations have important implications for provider education programs and development of intervention strategies for CFS.

## Background

Chronic fatiguing illnesses present diagnostic and management challenges for health care providers. Chronic fatigue syndrome (CFS) is a complex illness characterized by medically and psychiatrically unexplained disabling fatigue that is not relieved by rest and is accompanied by symptoms of prolonged postexertional malaise, unrefreshing sleep, impaired concentration and short-term memory, muscle or joint pain, headache, sore throat and tender lymph nodes [1]. In population-based studies of adults, the reported prevalence estimates for CFS appears to be between 0.24% [2] and 0.42% [3], and prevalence for CFS-like illness ranges from 0.25% [4] to 1.67% [5]. The illness is clinically challenging because, as yet, the etiology, pathophysiology and risk factors for CFS remain inchoate and treatment is targeted at treating symptoms and amelioration of consequences rather than providing a definitive cure.

Although not all persons suffering from CFS seek allopathic care [6,7], standard regimens of therapy include cognitive behavioral therapy, varying forms and regimens of exercise, and enhancement of coping skills, while caregiver prescribed or self-administered medication use remains common [8,9]. Recent increase in use of complementary and alternative medicine therapy (CAM) in various forms by the population at large [10-13] as provided by naturopaths, chiropractors, social workers, nutritionists, and acupuncturists, and others, has also been observed among patients with CFS [14-16]. Overall reports of CAM therapy use in subjects with a range of fatiguing illnesses have been based on clinical populations, have addressed the use of specific therapeutic modalities, or have reviewed the subject. The field lacks a systematic evaluation that addresses CAM use in fatiguing illnesses in the population at large, correlates of use, including demographic and geographic variables, presence of underlying illnesses, and standardized physical and mental health scores.

We examined the use of CAM categories and conventional medicine in a cross-sectional population survey of fatiguing illnesses in the United States and evaluated variables associated with their utilization Specifically we asked the following questions: (i) Do CFS-like and otherwise fatigued subjects use CAM and, if so, is it used more or less frequently than non-fatigued persons; (ii)Which modalities do they use; iii) What effects do demographic and geographic factors have on the association of CAM use and fatigue classification; (iv) Do the presence of accompanying/underlying illness and impaired health in general affect the use of CAM; (v) What conclusions can we make from the available data on CAM use by fatigued individuals that could be potentially useful to providers in their management of fatigued persons?

## Methods

This study adhered to human experimentation guidelines of the U.S. Department of Health and Human Services and the Helsinki Declaration. The CDC Human Subjects committee approved study protocols. All participants were volunteers who gave informed consent.

### Sampling Strategy

Data were collected during a random-digit dialing survey to estimate the prevalence of fatiguing illnesses in urban and rural populations from different geographic regions of the United States between July 2001 and January 2002 as detailed in Bierl et al.[5]. In brief, we used random digit dialing telephone survey to screen 2,728 randomly selected households. We conducted detailed telephone interviews of 455 persons between 18 and 69 years of age (76% participation), identified by the household informant (during the screening interview) as fatigued ≥ 1 month and 444 (74% participation) randomly selected persons identified as non-fatigued. Sampling included 8 geographic strata – Buffalo-Niagara Falls, New York (Northeast urban), Chicago, Illinois (Midwest urban), Baton Rouge, Louisiana (South urban), Oakland, California (West urban), Franklin County, Pennsylvania (Northeast rural), Ripley County, Indiana (Midwest rural), Monroe County, Georgia (South rural), and Chaves County, New Mexico (West rural). In each selected household, we screened an adult informant who enumerated individual household members and reported on their age, sex, race, and fatigue status. Informants were asked whether any household members were currently suffering from severe fatigue, extreme tiredness, or exhaustion that had lasted 1 month or longer.

### Data Collection

We conducted standardized detailed telephone interviews with household residents between 18 and 69 years of age who the informant identified as fatigued ≥ 1 month, and with a random sample of non-fatigued (NF) residents identified in the screening interview. The detailed interview [2,5] required approximately 30 minutes to complete and included questions on fatigue (duration of less than or greater than 6 months and whether it was relieved or not by rest), presence or absence of 19 symptoms during the preceding 4 weeks (8 specific for CFS and 11 associated with general illness), and symptom duration (less than or greater than 6 months). These data were used to classify subjects into subgroups of fatigued and NF participants. Information on demographics and history of pre-existing medical and psychiatric diagnoses were also collected and examined as covariates in our analyses. The interview also included the 12-item Short Form Health Survey (Quality-Metric, Inc., Lincoln, RI) that measures health related quality of life and functional impairment [17], which was also examined as a covariate. Finally, we obtained data on use of various CAM therapies in the past 12 months, and whether the subject discussed use of these therapies with their doctor, by administering the questionnaire shown in Table 1.

**Table 1:** Study Questions Concerning Use of CAM Therapies.

Did you discuss use of these treatments with your doctor?
Do you use or have you used any of the following forms of therapy in the preceding 12 months?
Acupuncture
Biofeedback
Chiropractic techniques
Commercial diet supplements
Energy healing, such as magnets, crystals, and energy emitting machines
Herbal and botanical supplements, such as ginseng, garlic, ginkgo biloba, echinacea, St. John's wort, or saw palmetto
High dose vitamins (not a daily multivitamin, or a daily calcium or iron supplement)
Homeopathy
Hypnosis
Imagery/visualization
Massage
Mindful exercise, such as yoga or tai chi
Personal prayer
Spiritual healing or prayer by others
Relaxation techniques, such as meditation
Self-help group, such as a 12 step program or support group
Are you using these approaches to treat and/or prevent a disease? Other treatments?
Do you think these approaches were useful?

### CAM Modalities and Categories

Since this study was formulated in 1999, we used as an operational definition the CAM modalities initially addressed by Eisenberg et al., [10] as assigned to 5 categories by Berman and Straus [18] including: 1) manipulative or body-based interventions including chiropractic and massage therapies; 2) alternative medical systems, such as homeopathy; 3) mind-body interventions including biofeedback, hypnosis, image/visual therapy, mindful exercise, personal prayer, prayer by others, relaxation techniques and self-help; 4) biologically-based therapies including diet supplements, herbal supplements, and vitamins; 5) energy therapies including acupuncture and energy healing. In addition we asked whether subjects discussed use of CAM therapies with providers, whether use was for treatment or prevention, and usefulness of the treatment(s) (Table 1).

### Classification of Study Participants

Individuals reporting fatigue lasting between one and 5-months were classified as having prolonged fatigue (PF); those with ≥ 6 months of fatigue were classified as having chronic fatigue (CF); and persons without fatigue for one month were considered as non-fatigued (NF). We classified chronically fatigued respondents as having a CFS-like illness if their fatiguing illness met criteria of the 1994 CFS research case definition [1]. In brief, criteria for CFS-like illness included chronic fatigue that was not alleviated by rest, and was accompanied by at least 4 of 8 symptoms (unusual post-exertion fatigue, impaired memory/concentration, unrefreshing sleep, headache, muscle pain, joint pain, sore throat, tender lymph nodes). The interview did not assess whether fatigue substantially interfered with work, educational, or personal activities, which is characteristic of CFS, nor did this study include a physical and psychiatric evaluation to confirm conditions that exclude classification as CFS. Fatigue categories were analyzed as discrete groups (NF, PF, CF and CFS-like), and each subject was counted only in the most applicable restrictive category.

### Statistics

This study analyzed the use of the 5 CAM categories (described above) by various groups of fatigued (CFS-like, CF, and PF) and NF subjects and examined the effect of various covariates on this relationship. For the purpose of analysis, race was categorized as White, Black and Other, geographic region was categorized as Northeast, South, West, or Midwest and rural and urban areas were analyzed separately. Education was categorized as <high school, high school graduate, some college or post-high school training, college graduate and post-graduate. Information on categorical household income was collected and treated as a dichotomous variable of ≤$40,000, >$40,000 based on the US median income. Physical and mental health scores, as measured by the SF-12, were treated as continuous measures. Other medical/psychiatric conditions were treated as counts in the analysis.

All statistical analyses were conducted in SAS version 9.0 (SAS Institute Inc, Cary, NC). We compared categorical variables between study groups by using either Chi-square or Fisher's exact tests and compared continuous variables between study groups by using either the T-test or the Wilcoxon rank-sum test. Correlations between continuous variables were computed using the Pearson Correlation Coefficient. We used separate multinomial logistic regression models (PROC GENMOD) to compute odds ratios (OR) as estimates of risk of CAM use among participants with various categories of fatiguing illness compared to the NF group. Dependent variables in these models included the following CAM categories: body-based, alternative medicine systems, mind-body including prayer, mind-body excluding prayer, biologically-based, and energy. All regression models included adjustment for age (continuous), sex, and count of other medical or psychiatric conditions. Additional covariates were included if they had p-values < 0.05 (income, education, geographic region). We further examined the effect of general physical and mental health on the adjusted ORs for the associations of fatigue case status and CAM categories by including the SF12 scores for physical and mental health in the regression models. We computed 95-percent confidence intervals (95% CI) to measure the precision of the ORs. We evaluated trend in ORs across a gradient of fatiguing illness categories by using separate logistic regression models for each CAM outcome and assigning values of 1–4 to a categorical variable representing study groups (1 = NF, 2 = PF, 3 = CF, 4 = CFS-like), adjusting for covariates. Wald test was used to calculate p-values for trend (p-trend). As is standard practice in regression models, subjects with missing values for any independent variable in a regression model were excluded from that specific analytic model.

## Results

### Subjects

Four hundred forty fatigued people and 444 non-fatigued (NF) respondents completed the full interview. Eligibility and refusal rates were similar for the two groups [5]. Fifty-three of the fatigued subjects were classified as prolonged fatigue (PF), 338 as chronic fatigue (CF), and 49 satisfied symptom criteria for CFS-like illness. These groups were similarly distributed by race, however they differed significantly by age and sex, with the PF group having a younger mean age and the NF group having a smaller proportion of women than other groups (p < .0001 for both). The NF group also reported a higher income than the three fatigue groups (p < 0.0001) (Table 2).

**Table 2:** Distribution of Demographic Factors by Illness Group

**Demographic Factor**	**CFS-like n = 49**	**Chronic Fatigue n = 338**	**Prolonged Fatigue n = 53**	**Non-Fatigued n = 444**	**p-value**
Age (mean ± SD)	42.5 ± 10.6	46 ± 12.2	38.5 ± 13.9	43.2 ± 13.5	p < 0.0001
					
Sex: n (%)					
Male	14 (28.5)	101 (29.9)	13 (24.5)	200 (45.0)	p < 0.0001
Female	35 (71.4)	237 (70.1)	40 (75.5)	244 (54.9)	
					
Race: n (%)					
White	44 (89.8)	294 (86.9)	43 (81.1)	369 (83.1)	p = 0.75
Black	3 (6.1)	24 (7.1)	8 (15.1)	46 (10.4)	
Other	2 (4.1)	20 (5.9)	2 (3.8)	29 (6.5)	
					
Income* (median): n (%)					
≤$40,000	21 (51.2)	176 (57.7)	33 (71.7)	158 (41.9)	p < 0.0001
>$40,000	20 (48.8)	129 (42.3)	13 (28.3)	219 (58.1)	
					
Education:** n, (%)					
< High school graduate	8 (16.7)	51 (5.8)	11 (20.7)	49 (11.4)	p = 0.19
High school graduate	17 (34.7)	140 (41.4)	19 (35.8)	150 (34.8)	
Some college	13 (27.1)	77 (8.8)	15 (28.3)	105 (24.4)	
College graduate	6 (12.2)	38 (11.2)	3 (5.7)	79 (18.3)	
Post graduate	4 (8.2)	27 (7.9)	5 (9.4)	48 (11.1)	

SD: standard deviation; *105 subjects were missing information on income;

**19 subjects were missing information on education

### CAM Use

Overall, 77% of participants reported using some CAM therapy in the 12-month period prior to the interview. Use of any CAM modality (i.e. ever in the preceding 12 months) was reported more frequently among women (56.8%) than men (44.2) (p = 0.0003), and increased significantly with increasing level of education (p-trend < 0.0001). Reported use of any CAM modality did not significantly vary by age, race, income or geographic regions (Table 3). Use of any CAM modality was more common (81.6%) among the combined groups of fatigued subjects than NF subjects (72.5%) (p=.0006). There were no striking differences in the proportion of participants who reported any CAM use between the PF, CF, and CFS like groups (79.3%, 82.3%, and 79.6%, respectively), but the mean number of CAM modalities used by each study group increased across subgroups of PF – CF – CFS-like. This trend was observed for women and men, but was statistically significant only among women (p = 0.03, p = 0.06 respectively).

**Table 3:** Distribution of Demographic Factors by Overall Use of Complementary and Alternative Medicine (CAM), excluding use of Prayer

**Demographic Factor**	**CAM Use**		**p-value**
		
	**No**	**Yes**	
Age (years)			p = 0.20
Mean ± st. deviation	43.9 ± 13.4	44.0 ± 12.7	

Sex: n, (%)			p = 0 .0003
Males	183 (55.7)	145 (44.2)	
Females	240 (43.2)	316 (56.8)	

Race: n, (%)			p = 0.36
White	356 (47.8)	391 (52.1)	
Black	43 (53.1)	38 (46.9)	
Other	21 (40.4)	31 (56.6)	

Income: n (%)			p = 0.45
≤$40,000	42 (47.2)	47 (52.8)	
>$40,000	163 (42.8)	218 (57.2)	

Education: n (%)			p = 0.0002
< High School	65 (54.6)	54 (45.4)	p-trend < 0.0001
High School	176 (53.9)	150 (46.0)	
Some College	93 (44.2)	117 (55.7)	
College graduate	48 (38.1)	78 (61.9)	
Post-graduate	26 (30.9)	58 (69.0)	

Geographic Region: n (%)			P = 0.85
Northeast	70 (25.1)	209 (74.9)	
Midwest	40 (22.9)	135 (77.1)	
South	56 (22.0)	198 (77.9)	
West	40 (22.7)	136 (77.3)	
			
Rural/Urban: n (%)			P = 0.39
Urban	91 (24.7)	277 (75.3)	
Rural	115 (22.3)	401 (77.7)	

The frequency of use of specific CAM therapies by study group is shown in Table 4. Except for each therapy in the biologically-based category, there was significant overall variation in the frequency of use of at least one of the specific CAM therapies within each of the remaining CAM categories (body-based, alternative medicine systems, mind-body, and energy) by illness group. In addition, within each of these CAM categories there was a significant trend of decreasing frequency of reported use across illness categories of CFS-like -CF-PF-NF for at least one CAM therapy.

**Table 4:** Frequency of Utilization of Complementary and Alternative Medicine Modalities According to Illness Classification

**CAM categories and specific modalities**	**CFS-like** ** (n = 48)**	**Chronic Fatigue** ** (n = 327)**	**Prolonged Fatigue** ** (n = 53)**	**Non-fatigued** ** (n = 435)**	**Overall p-Value**	**p-Trend**
	n (%)	n (%)	n (%)	n (%)	n (%)	

Body-based						
Chiropractic	12 (25.5)	57 (17.0)	10 (18.9)	39 (9.0)	.0004	.0001
Massage	17 (36.2)	71 (21.2)	1 (22.6)	65 (14.9)	.002	.001
						
Alternative Medicine System						
Homeopathy	7 (15.2)	30 (9.1)	1 (1.9)	26 (6.0)	.03	.02
						
Mind-body						
Biofeedback	3 (6.4)	8 (2.4)	1 (1.9)	6 (1.4)	.12	.06
Hypnosis	0 (0)	4 (1.2)	0 (0)	0 (0)	.11	--
Image/visual	7 (14.9)	39 (11.8)	9 (17.0)	34 (7.9)	.07	.04
Mindful exercise	4 (8.5)	27 (8.0)	5 (9.4)	23 (5.3)	.28	.11
Personal prayer	35 (74.5)	226 (67.5)	36 (67.9)	252 (58.1)	.01	.002
Prayer by others	23 (48.9)	114 (34.0)	12 (22.6)	92 (21.1)	<.0001	<.0001
Relaxation	11 (23.4)	90 (26.9)	10 (19.2)	60 (13.8)	.0001	<.0001
Self-Help	4 (8.5)	25 (7.5)	0 (0)	14 (3.2)	.01	.004
						
Biologically-based						
Diet supplements	8 (17.0)	47 (14.0)	2 (3.8)	50 (11.5)	.12	.17
Herbal supplements	13 (27.7)	75 (22.4)	10 (18.9)	80 (18.4)	.30	.07
Vitamins	5 (10.6)	28 (8.5)	5 (9.4)	28 (6.4)	.56	.27
						
Energy						
Acupuncture	1 (2.1)	8 (2.4)	2 (3.8)	5 (1.1)	.25	.21
Energy healing	4 (8.5)	24 (7.2)	2 (3.8)	16 (3.7)	.10	.02

Due to missing values, percentages cannot be computed based on total subjects

### Underlying Medical or Psychiatric Conditions

Overall, a significantly higher proportion of the combined group of fatigued participants (65.2%) reported having various medical or psychiatric conditions than the non-fatigued participants (38.5%) (p < 0.0001). None of these conditions were exclusionary according to the definition of CFS (1). The most commonly reported, but not verified, co-morbid diagnoses in the study population were: hypertension (13%), diabetes mellitus of unknown type (10%), thyroid disease (8%), asthma (8%), depression (7%), fibromyalgia (6%), hyperlipidemia (4%), cardiovascular disease (4%) and identifiable sleep disorders such as apnea and narcolepsy (<2%).

We examined the association of any CAM use with the presence of a medical or psychiatric condition. Among the NF and PF groups, similar proportions of those who did and did not report any CAM use had a medical/psychiatric condition (p = 0.19 and p = 0.68, respectively). However, among the CF group and CFS-like group, significantly higher proportions of those who reported any CAM therapy use had a medical/psychiatric condition, compared to those who did not use any CAM (p = 0.004, p = 0.0002, respectively). The mean number of medical/psychiatric conditions increased steadily across study groups, ranging from an average of 0.65 among the NF group to 1.8 among the CFS-like group (p < .0001).

We also explored whether use of CAM was associated with physical and mental health status, as measured by the SF-12. Non-fatigued subjects had scores comparable to published norms [17]. Since fatiguing illnesses can be defined in part by questions used in the Medical Outcomes Survey series [19], it is not surprising that the combined group of all fatigued subjects had significantly lower scores in both physical and mental health status compared to the NF group (p < .0001 for both). Interestingly, persons who reported using any of the 5 CAM categories had significantly poorer physical health scores (i.e. lower median SF-12 scores) than persons who did not report using CAM therapies (Table 5). In addition, users of all but the alternative medicine system, homeopathy, had significantly poorer mental health scores than persons who did not use these CAM categories or conventional medicine.

**Table 5:** Distribution of Median Scores for the SF-12 Physical and Mental Health Subscales, by Category of CAM Use

**CAM Category/Conventional Use**	**Physical Health Median Score (range)**	**Mental Health Median Score (range)**
Body-based		
Yes (n = 228)	44.8 (14.1 – 62.4)	46.3 (17.8 – 66.4)
No (n = 623)	50.4 (12.1 – 64.9)	50.6 (14.2 – 67.6)
	(p < 0.0001)	(p = 0.01)

Alternative Medicine		
Yes (n = 64)	44.9 (18.8 – 60.1)	44.1 (19.1 – 64.7)
No (n = 787)	49.6 (12.1 – 64.9)	49.7 (14.2 – 67.6)
	(p = 0.04)	(p = 0.14)

Mind-body (excluding prayer)		
Yes (n = 219)	44.6 (12.1 – 64.9)	44.6 (17.8 – 66.4)
No (n = 632)	50.8 (12.7 – 64.6)	50.6 (14.2 – 67.6)
	(p < 0.0001)	(p < 0.0001)

Mind-body (including prayer)		
Yes (n = 579)	48.1 (12.1 – 64.9)	47.3 (14.6 – 66.4)
No (n = 272)	51.4 (15.8 – 61.4)	52.5 (14.2 – 67.6)
	(p = 0.001)	(p < 0.0001)

Biologically-based		
Yes (n = 220)	48.3 (12.1 – 64.9)	44.6 (14.6 – 66.4)
No (n = 631)	49.6 (12.7 – 64.6)	50.3 (14.2 – 67.6)
	(p = 0.36)	(p < 0.0001)

Energy		
Yes (n = 59)	41.9 (18.0 – 60.6)	43.2 (19.1 – 63.4)
No (n = 792)	49.7 (12.1 – 64.9)	49.9 (14.2 – 67.6)
	(p = 0.003)	(p = 0.001)

p-values are based on the Wilcoxon test

SF-12 norms by age group: 18–44 years = Physical 52.5, Mental 48.4; 45–55 years = Physical 50.2, Mental 50.8

### Discussion of CAM Use with Providers

Overall, 37.5% of all respondents said that they discussed their CAM therapy use with their physician, but this practice increased across subgroups, ranging from a frequency of 26.1% among the NF subgroup to 57.6% among the CFS-like group (p-trend = 0.0001). Individuals previously diagnosed with CFS reported more CAM therapy use than other persons with fatigue who were not diagnosed with CFS.

### Model of Association of CAM Use with Fatiguing Illnesses

Odds ratios for use of body-based therapy increased in a significant trend across fatigue categories of PF, CF and CFS-like subjects (p-trend = .0003) (Table 6). Notably, the CFS-like group had a greater than 3-fold increased likelihood of using body-based therapy (OR = 3.82, CI = 1.94, 7.51) than the NF group, after adjusting for age and sex. However, after further adjustment for number of other diagnosed medical and psychiatric conditions reported by participants, the association of body-based therapy with the CFS-like group was diminished, but remained statistically significant (OR = 2.52, CI = 1.32, 4.82), as did the trend across study groups (p = 0.002). Body-based therapy was also significantly more likely to be used by the CF group compared to the NF group (OR = 1.52, CI = 1.07, 2.16). Use of body-based therapy was also independently and significantly associated with female sex, high income and higher number of other diagnoses (p = 0.002, p = 0.003, p = 0.03, respectively).

**Table 6:** Adjusted Odds Ratios (OR) and 95% Confidence Intervals (CI) for Associations of Fatigue Groups with Use of Complementary and Alternative Medicine Categories

	**CFS-like**	**Chronic Fatigue**	**Prolonged Fatigue**	**Non-fatigued**	**p-trend**
**CAM use**	**OR 95% CI**	**OR 95% CI**	**OR 95% CI**	**OR**	

Body-Based^†^	2.52 (1.32–4.82)	1.52 (1.07–2.16)	1.67 (0.88–3.17)	1.0	0.002
Alternative Medicine^+	2.55 (0.97–6.71)	1.43 (0.79–2.59)	0.29 (0.04–2.22)	1.0	0.07
Mind-body (includes Prayer)^	1.33 (0.63–2.83)	1.17 (0.83–1.66)	1.39 (0.70–2.73)	1.0	0.28
Mind-body (excludes Prayer)^	1.47 (0.73–2.97)	1.73 (1.20–2.48)	1.65 (0.84–3.22)	1.0	0.006
Biologically-based^	1.48 (0.77–2.87)	1.19 (0.84–1.68)	0.92 (0.46–1.81)	1.0	0.19
Energy	1.86 (0.65–5.38)	1.58 (0.86–2.91)	1.54 (0.50–4.74)	1.0	0.09

All models include adjustment for age (continuous) and sex and count of other diagnoses; ^† ^includes adjustment for income; ^ includes adjustment for education; +includes adjustment for geographic area

The CFS-like group was also 2.9-times more likely to report using an alternative medical system (represented by homeopathy) compared to the NF group after adjusting for age, sex, education and census region (OR = 2.91 CI = 1.14, 7.40); there was also a significant trend of decreasing ORs associated with homeopathy use across study groups (p-trend < .0001) (Table 6). After further adjusting for number of other diagnoses, the OR associated with homeopathy use among persons with CFS-like illness was attenuated and no longer statistically significant (OR = 2.55, CI = 0.96, 6.71). The trend associated with homeopathy use by the fatigue subgroups was also no longer statistically significant (p = 0.07). However, use of homeopathy was independently and significantly associated with female sex, higher level of education and geographic area, with use reported more frequently in the West region of the U.S. (p = 0.007, p < .0001 p = 0.03, respectively).

Mind-body CAM use was not reported significantly more often by any of the fatigue groups compared to the NF group (Table 6). However, personal prayer and prayer by others are considered mind-body therapies and are commonly practiced by all study groups. After removing personal prayer and prayer by others from the mind-body category (which left image/visual therapy, mindful exercise, relaxation techniques), use of any of these remaining mind-body therapies was 73% more likely among the CF group compared to the NF group (OR = 1.73, CI = 1.20–2.48), and there was a significant trend of decreasing likelihood of use across study groups of CFS-like – CF-PF – NF (p-trend = .006). In addition, use of such mind-body therapies was independently and significantly associated with females sex, higher education and higher number of other diagnoses (p = 0.003, p < 0.0001, p = 0.001, respectively). None of the fatigue subgroups were significantly more likely to use biologically based therapies than the NF group; however, use of biologically based therapies was significantly associated with higher education and a higher number of other diagnoses (p < 0.0001, p = 0.002).

We further examined whether the associations of body-based CAM and mind-body (excluding prayer) CAM use with CFS-like illness and chronic fatigue, respectively, could be accounted for by physical or mental health. SF-12 scores for physical health and mental health were significantly but weakly correlated (r = 0.15, p < 0.0001), allowing for their simultaneous addition to the fully adjusted models predicting use of these CAM categories. Physical health remained significantly and independently associated with use of body-based CAM (p = 0.002) after consideration of illness group and other covariates. Mental health remained significantly and independently associated with use of the mind-body CAM (excluding prayer) (p = 0.003). However, after accounting for the effects of physical and mental health scores, the association between CFS-like illness and body-based CAM use was attenuated and no longer statistically significant (OR = 2.05, CI = 0.96 – 4.39). The association of CF illness and mind-body (excluding prayer) CAM was no longer detected (OR = 0.91, CI = 0.59 – 1.42).

## Discussion

Our study addressed the use of complementary and alternative medicine (CAM) therapies identified in the 1990's by persons with fatiguing illnesses and non-fatigued persons. As defined here, 77.0% of subjects reported CAM use between July 2001 and January 2002, a greater proportion than that previously reported at the national level, where in general it ranged from 36% in 1990, to 46% in 1997, and 62% in 2002 [10-13]. The obvious difference between the current report and previous observations is the inclusion of subjects with fatiguing illnesses in this report, 81.6% of whom reported CAM therapy use versus 40% use by those with specific illnesses in the 2002 study (13). However the non-fatigued subjects in our study reported an overall 72.5% prevalence of CAM therapy use. The less obvious difference in prevalence observed here is related to the exclusion of analyses of personal prayer in earlier published reports. Personal prayer was used more than any other CAM in our study; 63.1% of our study subjects reported its use compared to 43% in the general population in 2002. Our results are reminiscent of data from 1999 National Health Interview Survey [20], which found that prayer and herbs were the most common CAM therapies used, with the greatest use among those with self-described poor health.

Compared to the non-fatigued group, persons classified as having CFS-like illness and those classified as having chronic fatigue were significantly more likely to use only the body-based CAM therapy category, which includes chiropractic and massage therapies. We also found that compared to the NF group, frequency of use of body-based therapy by subjects with fatigue increased across groups of PF, CF and CFS-like in a linear trend.

After excluding use of personal prayer and prayer by others, use of other components of the mind/body category was reported significantly more often by the chronic fatigue group compared to the non-fatigued group. However, the ORs associating use of these and other CAM categories were substantially attenuated when we adjusted for the number of other, non-exclusionary medical/psychiatric diagnoses. Further adjustment for physical and mental health, as measured by scores on the SF-12, further diminished these associations and resulted in their non-significance. Taken together, these data suggest that both the presence of "other diagnoses" and poorer physical and mental health account for some of the use of body-based and mind-body CAM therapies by persons with CFS-like illness and CFS.

Pearson et al. [21] identified insomnia, hypertension, congestive heart failure, anxiety or depression, and obesity, as entities leading to the use of CAM therapies. In principle, our data support this conclusion, since hypertension, depression, and conditions known to be risk factors for congestive heart failure (hyperlipidemia, diabetes mellitus) were among the common underlying medical conditions in our study. Insomnia, in the absence of diagnosable sleep disorders, and as defined by Pearson et al., [21], however, is included in the CFS construct and could not be considered as a separate underlying condition. In practice we did not specifically address the question of whether CAM therapy was used for the fatiguing illnesses that were identified or for an underlying disease.

In the general population, CAM therapies were used as adjuncts to standard care, health promotion, or disease prevention [10]. Although we asked participants whether they used CAM for treatment or prevention of illness, we question the validity of this measure based on the ambiguous response categories and did not include it in our analysis. However, a higher proportion of subjects in the CFS-like (57.5%) and CF (47.9%) groups discussed their use of CAM with their primary providers than the non-fatigued CAM users (26.1%), suggesting that persons with fatiguing illnesses use both allopathic and alternative medical care. Further analysis of subject's rationale for use of CAM and the relationship to satisfaction with allopathic care is necessary in order to determine if CAM therapies are used in deference to standard care [22].

Interpretation of our results must take into consideration the limitations of this study. We classified subjects based on telephone interviews with the inherent problems of under-representation of low income, and transient populations, and did not conduct in-person interviews, physical examinations or laboratory tests to rule out other conditions associated with fatigue. It is known that approximately 20% of those diagnosed with CFS or CFS-like illness have other conditions that are exclusionary for the diagnosis of CFS [3], so it is likely that our CFS-like group includes persons who would have been excluded had we conducted further screening tests. Thus our results cannot be generalized to CFS, but rather, would be useful to physicians and other health care providers whose practices include approximately 25% of patients with unexplained fatiguing illnesses. In addition, our analysis involved comparisons of multiple CAM categories between 3 fatigued groups and a NF group, increasing the probability of finding an association by chance. However, after adjusting for covariates that included physical and mental health, as well as other medical and psychiatric conditions, none of the associations of increased CAM use by the CFS-like or CF groups remained significant, making adjustment for multiple comparisons unnecessary.

On a practical note, physicians see and classify these patients and must know about their use of CAM in order to plan or modify their evaluation, diagnosis, and treatment strategy. Understanding the patients' rationale for CAM therapy and their perception of its efficacy may provide information on whether symptoms or the illness dictate intervention and on positive or negative consequences of CAM therapy [23,24]. Further information on the use of prayer and its efficacy in treating illnesses, as well as the place of prayer in studies of CAM therapy are needed. This issue should be examined within the context of general health status and cultural and religious practices [25].

## Conclusion

CAM use in fatiguing illness is more prevalent than it is in non-fatigued persons. Body-based therapies (chiropractic and massage therapy) and mind-body (excluding prayer) therapies were significantly more likely to be used by persons with chronic fatigue or CFS-like illnesses, compared to non-fatigued controls. However, poorer physical and mental health and the presence of other physical and psychiatric conditions, appear to account for greater use of these CAM therapies by persons with chronic fatigue and CFS-like illness.

## Competing interests

The author(s) declare that they have no competing interests.

## Authors' contributions

JFJ, ABJ, and WCR designed the study. JFJ, ABJ, EM, and RB identified the questions to be analyzed in the report. EM and RB performed the statistical analyses. All authors contributed to writing the report and have read and approved the final manuscript.

## Pre-publication history

The pre-publication history for this paper can be accessed here:


